# Screening: the information individuals need to support their decision: per protocol analysis is better than intention-to-treat analysis at quantifying potential benefits and harms of screening

**DOI:** 10.1186/1472-6939-15-28

**Published:** 2014-03-28

**Authors:** Paolo Giorgi Rossi

**Affiliations:** 1 Servizio Interaziendale di Epidemiologia, AUSL Reggio Emilia, Via Amendola 2, Reggio Emilia I42122, Italy

**Keywords:** Mass screening, Informed consent, Participation, Informed choice, Intention to treat analysis, Per protocol analysis, Systematic reviews, Consumer health information, Health literacy

## Abstract

**Background:**

Providing individuals with the information necessary to make informed decisions is now considered an ethical standard for health systems and general practitioners.

**Discussion:**

Results from intention-to-treat analysis have thus far been used to illustrate screening benefits and harms, but intention-to-treat analysis in most screening trials compares no intervention to invitation to screening. Therefore, the intervention arm includes everyone who was invited, regardless of actual participation. These results may be misleading for individual decision-making. We propose to use a per protocol analysis that includes all subjects who presented to screening and compares them to those in control arm, adjusting for self-selection bias. Such an analysis can give more accurate and useful information for individual decision-making.

**Summary:**

Correct information for individual decision to participate in screening or not should consider the efficacy, benefits, and harms observed for subjects who actually participated at least once in screening compared to the control arm, adjusting for self-selection bias. Thus, per protocol analysis, even a very conservative one, should be used, not a full intention-to-treat analysis.

## Background

Providing individuals with the information necessary to make informed decisions is now considered an ethical standard that screening programs and opportunistic secondary prevention must meet [1-3]. The general public must be aware of the benefits of screening, i.e. mortality reduction and, for cervical and colorectal cancer, reduced incidence, but also of its harms, i.e. overdiagnosis and unnecessary workup and treatment.

To this end, several systematic reviews have recently summarised the results of numerous randomised controlled trials (RCTs) on the benefits and harms of screening. These summaries provide pooled relative risk estimates as well as absolute numbers of events occurring in a cohort of screened individuals compared to what would occur in a cohort of unscreened individuals, based on intention-to-treat (ITT) analysis [4-6]. Presenting the results in terms of absolute numbers in a small cohort is the most effective way to provide correct information on the benefits and harms of a procedure: numerical information is exact, straightforward (thereby reducing cognitive complexity), and extremely concrete (thus easy to imagine) [7,8]. However, these numbers, as we will see, may not be the most appropriate choice to support informed decision-making.

Most of the screening systematic reviews used ITT analysis to calculate the expected number of events (benefits and harms) in the screened and non-screened cohorts, which is a standard choice of analysis. For policy makers, intention-to-treat analysis - a weighted average of harms and benefits occurring in participants and non-participants alike - is the most useful since it measures the actual impact that a screening programme would have at the population level, taking into account also that the most important barrier to effectiveness is often non-participation [9,10].

Most of the trials included in the systematic reviews that demonstrated screening efficacy (prostate, breast, and colorectal screening) were designed to randomise the target population to intervention (invitation to screening) or to no intervention (control – no invitation: for prostate cancer screening both PLCO [11] and ERSPC trials [12]; for breast cancer screening all the Swedish trials, Malmo, Goteborg, Stockholm, Two counties, the UK age trial, New York HIP trial, and Edinburgh. For references see the Cochrane systematic review [13] or the UK Independent Panel [3]; for colorectal cancer most of the trials on faecal occult blood test, Funen, Goteborg, Minnesota, and Nottingham. For references see the Cochrane systematic review [14]).

In the context of screening, however, intention-to-treat analysis results may not actually support individual decision-making. If the ITT analysis results from these RCTs are used to that end, we are providing the general public with information based on the comparison of the population randomised to receive the screening invitation with the population who did not receive the invitation. This information, in other words, examines the benefits and harms of screening between those who received the invitation and those who did not, and not the harms and benefits of actually participating in screening. The individual who must decide whether or not to participate in a screening programme, therefore, may be mislead by the very information intended to support him/her. This problem could be resolved by using a per protocol (PP) analysis, which compares those individuals who actually participated, i.e. at least presented for the first screening test, with those who were not invited. A recent review showed that most of the differences in the estimates of mortality reduction due to mammographic screening disappear when the figures are calculated only for women who were actually screened [15].

## Discussion

Table 1 illustrates the kind of information provided to the general public on breast cancer screening: three plain language syntheses or fact boxes produced for individual decision-making on breast screening based on recent systematic reviews. These figures were produced by the UK Independent Panel [3], by the Euroscreen Working Group [4], and by the Harding Centre for Risk Literacy [5]. All three provide ITT estimates on the benefits of screening; surprisingly, the full papers by The UK Independent Panel and by Euroscreen also report per protocol estimates (women who actually responded). As for the harms of screening, the UK Independent Panel fact sheet clearly states that the estimate of overdiagnosis is calculated for women actually screened “as a proportion of cancers detected during their screening period”. Understanding the fact sheets is difficult, which defeats their purpose. Further, their plain language/narrative presentation does not clarify which estimates are used and to which population, invited or screened, they apply. Individuals may believe they have understood the information and have made an informed choice, but have they?

**Table 1:** Comparison of three plain language syntheses or fact boxes summarising benefits and harms of mammographic screening for breast cancer as reported by the authors (intention-to-treat, ITT) and re-computed according to an individual decision making perspective (per protocol, PP)

	**UK independent panel**[4] 10,000 women aged 50 screened every three years for 20 years			**Euroscreen group**[5] 1,000 women aged 50 screened every two years for 20 years and followed up for 30 years			**Harding Centre**[6]**, Based on Nordic Cochrane review**[13] 2,000 women >50 yrs screened for 10 years		
**Population time frame**									
	**ITT**		**PP**	**ITT**		**PP**	**ITT**		**PP**
	**Invited**	**Not invited**	**Screened**	**Invited**	**Not invited**	**Screened**	**Invited**	**Not invited**	Screened*
Cancer specific deaths	-	-	-	21-23	30	21-23	7	8	6.6
Prevented deaths	43	-	56	7-9	-	12-14	1		1.4
Cancer incidence	681	552	720	71	67	73			
Additional cancer	129	-	168	4	-	6	10		14.2
Ascertainments				100		143			
Non invasive				70		100			
Invasive				30		43			
Psychological distress							200		286

*For the Harding Centre fact sheet the per protocol figures were estimated by applying the average participation rate used by the Euroscreen group [4].

To illustrate the difference between intention-to-treat analysis results and per protocol estimates, we can use the same approach as the Harding Centre for Risk Literacy for mammography, applied to colorectal screening. With ITT, we obtain an estimate for colorectal cancer screening with faecal occult blood test of 85 deaths out of 10,000 persons screened for ten years and 100 deaths for the same population in the absence of screening. With a per protocol analysis, there would be 74 deaths out of 10,000 persons screened with at least one test in 10 years.

Which should the individual take into consideration in the decision-making process, 85 deaths per 10,000 screened or 74 deaths per 10,000 screened?

The Cochrane Review on colorectal screening does take these two calculations into account, however; both the mortality reduction for the whole randomised population and adjusted for participation are reported [14].

The first issue to be addressed concerns using ITT analysis results for the purpose of helping individuals invited to screening to decide whether or not to participate. Indeed, doing so would result in the paradox that the invitee would apparently be affected by any of the benefits and/or harms observed in the ITT analysis thanks solely to having received the invitation, regardless of actual participation. Clearly, however, both harms and benefits are downstreams of screening participation. Therefore, the correct numbers to use to help inform individual decision-making are those regarding the subjects who actually participated, which do not come from the ITT analysis but from PP.

Intention-to-treat remains the first and only analysis to test the hypothesis for the overall effectiveness of any intervention; rejection of the null hypothesis in the intention-to-treat analysis is a prerequisite to proceed to per protocol analysis.

In the case of a therapeutic intervention in the clinical setting, most of the causes of therapy interruption or non-compliance to the protocol are both prognostic factors and causally linked to the therapy itself (intolerance, side effects, etc.). The best prospective estimate of therapy efficacy that we can therefore give patients who must chose between therapeutic options is based on intention-to-treat analysis, given that it cannot be known *a priori* whether a therapy will be tolerated and/ or finished.

An individual’s decision to participate in screening, however, is not usually determined by potential negative or positive prognostic factors, and there is no causal link between participation and screening results. Therefore, if participants and non-participants differ in their baseline mortality or incidence this will be due to a self-selection bias.

Further, the target screening population is healthy; in the absence of other interventions, it is thus absolutely unlikely that a large number of deaths or cancer diagnoses will occur in the time elapsing between invitation and participation. Therefore, any potential bias that per protocol analysis may introduce due to the exclusion of outcomes that occurred between randomization and screening test should not be relevant, while it can be quite relevant when the mortality rate in the study population is high, i.e. in many therapeutic trials [16].

**Figure 1:**
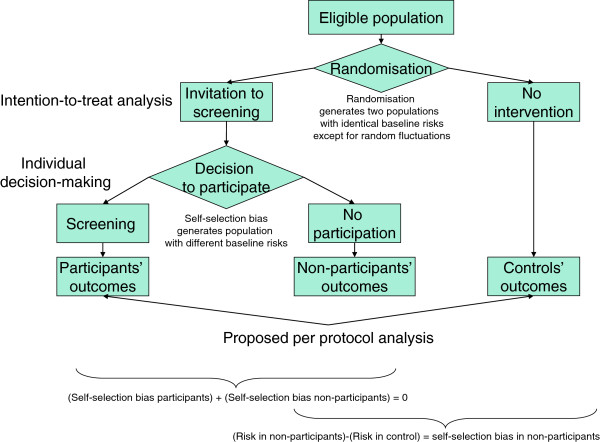
Theoretical framework of the intention-to-treat and the proposed per protocol analysis applied to cancer screening trials that randomised to invitation to screening or no intervention.

Given the absence of any causal link between participation and screening results (the decision is made before the first screening test) and given the data produced by the trials, the self-selection bias mentioned above can be adjusted for. In fact, we can measure incidence and mortality in the controls and compare them with those of the non-participants, thereby obtaining a direct measure of the self-selection bias (Figure 1). This measure is based on the outcomes themselves and thus includes all the possible effects of detected and undetected confounding variables.

In conclusion, a correct per protocol analysis can produce data useful to support individual decision-making providing it meets the following conditions: 1) it must include all the randomised subjects who *presented* for the test; 2) it must not exclude any subject for any reason after presentation; 3) it must compare the results in this cohort with those of the control arm; 4) it must appropriately adjust for self-selection bias. The conceptual flow chart of this strategy is represented in Figure 1.

A similar framework could be applied to estimate benefits and harms of other preventive interventions in which trials are designed to measure the impact on population while the impact on individuals depends on participation in the intervention itself.

## Summary

Providing individuals with the information necessary to make informed decisions is now considered an ethical standard for health systems and general practitioners.

Results from intention-to-treat analysis have thus far been used to illustrate screening benefits and harms.

Intention-to-treat analysis in most screening trials compares no invitation (control) to invitation to screening (intervention). The intervention arm therefore includes everyone who was invited, regardless of actual participation. These results may be misleading for individual decision-making.

Correct information should consider the efficacy observed for subjects that participated at least once in screening compared to the control arm.

## Abbreviations

ITT: Intention to treat; PP: Per protocol.

## Competing interests

The author declares that he has no competing interests. Non financial competing interests: the author is involved in several projects to increase appropriateness in diagnostic test use and in evaluating the impact of organised screening programs.

## Pre-publication history

The pre-publication history for this paper can be accessed here:

http://www.biomedcentral.com/1472-6939/15/28/prepub
